# Death after smoking of fentanyl, 5F-ADB, 5F-MDMB-P7AICA and other synthetic cannabinoids with a bucket bong

**DOI:** 10.1007/s11419-023-00666-w

**Published:** 2023-06-10

**Authors:** Merja A. Neukamm, Sebastian Halter, Volker Auwärter, Georg Schmitt, Arianna Giorgetti, Marc Bartel

**Affiliations:** 1 Institute of Forensic Medicine, Forensic Toxicology, Medical Center - University of Freiburg, Faculty of Medicine, University of Freiburg, Albertstrasse 9, 79104 Freiburg, Germany; 2 Institute of Forensic and Traffic Medicine, University Hospital, Voßstrasse 2, 69115 Heidelberg, Germany; 3 Department of Medical and Surgical Sciences, Unit of Legal Medicine, University of Bologna, Via Irnerio 49, 40126 Bologna, Italy

**Keywords:** Lethal intoxication, Cannabimimetic, Drugs of abuse, Fatality, Opioids, New psychoactive substances (NPS)

## Abstract

**Purpose:**

We report a case of a polydrug user who consumed various synthetic cannabinoids and fentanyl from a transdermal patch via a bucket bong. Toxicological results from postmortem matrices with special focus on synthetic cannabinoids are discussed in terms of their relevance to the death.

**Methods:**

The samples were analyzed by toxicological screening procedures involving immunoassays and gas chromatography–mass spectrometry (GC–MS) as well as quantitative analyses by means of GC–MS and high-performance liquid chromatography–tandem mass spectrometry (LC–MS/MS).

**Results:**

At the autopsy, coronary artery disease and signs of liver congestion were noted, in the absence of acute myocardial ischemic changes. Femoral blood concentrations of fentanyl and pregabalin were 14 ng/mL and 3,200 ng/mL, respectively. In addition, 2.7 ng/mL 5F-ADB and 13 ng/mL 5F-MDMB-P7AICA were detected together with relatively low amounts of 5 other synthetic cannabinoids in cardiac blood. A total number of up to 17 synthetic cannabinoids were detected in kidney, liver, urine and hair. Fentanyl and 5F-ADB were also detected in the water of the bucket bong.

**Conclusions:**

The cause of death could be attributed to an acute mixed intoxication by fentanyl and 5F-ADB (both Toxicological Significance Score (TSS) = 3) with a contribution of pregabalin and 5F-MDMB-P7AICA (TSS = 2), in a subject suffering from pre-existing heart damage. The most plausible mechanism of death consists in a respiratory depression. This case report demonstrates that use of opioids in combination with synthetic cannabinoids might be particularly dangerous.

## Introduction

Smoking drugs of abuse can lead to a rapid onset of drug effects and high concentrations in the central nervous system. In the case of drugs like opioids or synthetic cannabinoids, this can lead to fatal outcome even in individuals which developed tolerance to these drugs. Acute toxicity can be additionally elevated when the drug is inhaled in a highly efficient way, e.g. by smoking a bucket bong. Smoking a bucket bong results in a high concentration of the active ingredient in the lungs, because dense smoke can be forced into the lungs quickly.

Synthetic cannabinoids are a group of drugs pertaining to the class of new psychoactive substances (NPS) that mimic the effects of THC and are typically consumed by smoking. They are known to produce a feeling of being “stoned” or “high” but could lead to acute severe intoxications and even death. Synthetic cannabinoids have been linked to arrhythmia, acute kidney injury, seizures and psychiatric complications, as well as to respiratory depression [1, 2]. For 5F-ADB (methyl 2-{1-(5-fluoropentyl)-1*H*-indazole-3-carbonyl] amino} 3,3-dimethylbutanoate) in particular psychomotor agitation, impaired consciousness, anxiety, seizures, tachycardia and acute circulatory failure after drug inhalation were described as symptoms of intoxication or causes of death [2–5]. As for other compounds, tolerance to synthetic cannabinoids can be induced by frequent use [6, 7].

Fentanyl is a synthetic anesthetic and pain reliever, also used for recreational purposes, which has a higher pharmacological potency than heroin and morphine (50–100 times stronger). After consumption of fentanyl, heroin users report overcoming tolerance to opioids or the inhibitory effect of antagonists [8]. Typical symptoms of fentanyl overdoses are central nervous system (CNS) depression with lethargy until coma and respiratory arrest [9]. Fentanyl for non-medical use can e.g. be obtained in the form of used transdermal patches, as discarded fentanyl patches still contain 28–84% of the original dose after three days of therapeutic use [10]. The liquid extracted from patches can then be used for injection or volatilized and inhaled [11]. Synthetic opioids like fentanyl could as well be ordered as pure substance via shops in the Internet [12].

We report the fatal case of a polydrug user, who used various synthetic cannabinoids along with fentanyl by inhalation via a bucket bong. The toxicological results from postmortem samples are presented with particular emphasis on synthetic cannabinoids and discussed in terms of their relevance to the cause of death.

## Case history

**Fig. 1:**
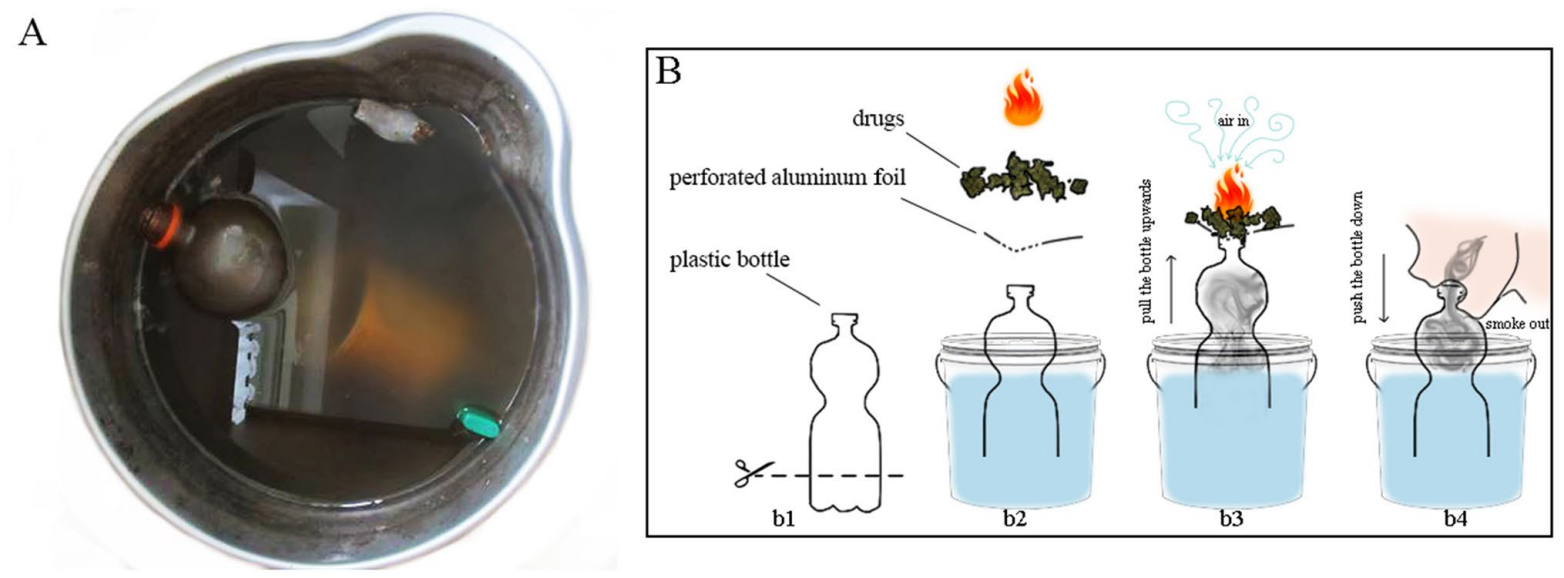
**A** Bucket with water and immersed bottle found at the scene. **B** Scheme of smoking drugs with a bucket bong. Bottle is cut (b1), bottle is immersed deep into water (b2), smoke is produced by air flow through burning drug (b3), smoke is pushed out and inhaled (b4)

In the present case, witness reports and exhibits at the death scene indicated drug consumption with a so-called ‘bucket bong’ or ‘gravity bong’, a method for smoking drugs (Fig. 1A). To smoke a ‘bucket bong’, the top cut off of a plastic bottle (here: 1.5 L soft drink bottle) is usually placed in a bucket full of water until the water covers the neck of the bottle. The opening of the bottle is then covered with perforated aluminum foil, and the drug or preparation is ignited on the foil. The bottle is then slowly pulled upwards until only a bottom part of the bottle remains in the water. This causes an air flow through the burning drug and releases dense smoke into the bottle. Then, the aluminum foil is removed and the smoke is inhaled through the bottle opening by pushing the bottle down into the bucket (Fig. 1B). This procedure results in a high concentration of the active ingredient in the lungs, as the smoke is forced into the lungs quickly and with pressure. The relatively high drug concentration in the pulmonary arteries and the bloodstream can lead to a fast onset of strong effects compared to oral consumption and even smoking of a cigarette mixed with the drugs.

In the present case, a 35-year-old man was found kneeling over a bucket with his head just above the water. Emergency medical services were notified by his friends, but resuscitation attempts were unsuccessful. Another man, who had been using drugs together with the deceased that day, survived. According to witnesses, both men would regularly use drugs like ‘herbs’ and opioids in a mixture via a ‘bucket bong’. For this purpose, a small part of a prescribed fentanyl transdermal therapeutic system (TTS) would be crushed and mixed with the ‘herbs’. The deceased was registered by the police as a drug user and for trafficking synthetic cannabinoids.

## Materials and methods

### Postmortem examination and sampling

The autopsy was performed 4 days after death and included a complete postmortem examination as well as collection of fluids and biological material for histological and toxicological analyses. Particularly, peripheral blood (femoral vein blood), central blood (cardiac blood), urine, gastric content, hair as well as samples from organs were collected for chemical toxicological investigations.

### Initial toxicological screening

Femoral vein blood, cardiac blood and urine, collected at the autopsy, were stored frozen at − 20 °C until analysis. Volatile substances including ethanol were determined in femoral blood by headspace gas chromatography as well as by the alcohol dehydrogenase method after deproteinization. Urine was screened for drugs of abuse by immunoassays. Urine and gastric contents were also screened for basic, neutral, and acidic drugs by comprehensive general unknown screening with gas chromatography–mass spectrometry (GC–MS). The urine sample was not hydrolyzed or derivatized prior to analysis.

### Quantitative analyses

Fentanyl and norfentanyl were quantified in femoral and cardiac blood by high-performance liquid chromatography-tandem mass spectrometry (LC–MS/MS) after liquid–liquid extraction. Pregabalin was quantified in femoral blood by LC–MS/MS after deproteinization. After analysis of fentanyl and pregabalin, the remaining volume of femoral blood was too low for analysis of tetrahydrocannabinol (THC) and metabolites and synthetic cannabinoids. THC and metabolites were quantified in cardiac blood by GC–MS after liquid–liquid extraction. Synthetic cannabinoids were quantified in cardiac blood, kidney, liver and hair by LC–MS/MS. Qualitative analysis for synthetic cannabinoid metabolites was performed in urine by LC–MS/MS after liquid–liquid extraction.

### Chemicals, reagents and standards

Initial toxicological screening: ethanol standard solutions were supplied from Diasys Diagnostic Products (0.5, 1.0, 2.0, 3.0, and 4.0 mg/mL; Holzheim, Germany) and Medichem (0.2 mg/mL; Steinenbronn, Germany). Bi-distilled water was from B. Braun (Melsungen, Germany). Acetonitrile (ACN), methanol, *tert*-butanol, isopropanol, chloroform, methylene chloride, trichloroacetic acid were purchased from Carl Roth (Karlsruhe, Germany).

Quantitative analyses: Methanol (HiPerSolv CHROMANORM^®^) and ACN (HiPerSolv CHROMANORM^®^) for LC–MS/MS were purchased from VWR Chemicals (Radnor, Pennsylvania, USA). ACN, methanol, *tert*-butanol, isopropanol, chloroform, methylene chloride, trichloroacetic acid were from Carl Roth (Karlsruhe, Germany). Ethyl acetate (p.a.) was obtained from Honeywell Riedel-de Häen^®^ (Seelze, Germany). Sodium hydrogen carbonate (≥ 99.5%, anhydrous) and formic acid (p.a.) were from Carl Roth GmbH (Karlsruhe, Germany). Ammonium formate was obtained from Sigma-Aldrich (St. Louis, Missouri, USA). Sodium carbonate (≥ 99.5%, anhydrous) and *n-*hexane (LiChrosolv) were purchased from Merck (Darmstadt, Germany). Pure water Ampuwa^®^ was from Fresenius Kabi (Bad Homburg, Germany). Deionized water was prepared using a Medica^®^ Pro deionizer from ELGA (Celle, Germany). Deuterated methanol (CD_3_OD) was obtained from Euriso-top (Saint-Aubin, France).

Delta-9-THC, delta-9-THC-D3, 11-nor-9-carboxy-delta-9-THC-D3, 11-hydroxy-delta-9-THC, 11-hydroxy-delta-9-THC-D3, 11-nor-9-carboxy-delta-9-THC, cocaine, cocaine-D3, benzoylecgonine, benzoylecgonine-D3, fentanyl, fentanyl-D5, norfentanyl, norfentanyl-D5, pregabalin, vigabatrin were purchased from LGC (Wesel, Germany). 5F-ADB (5F-MDMB-PINACA), AB-FUBINACA, ADB-FUBINACA, AMB, EG-018 and FUB-AMB (AMB-FUBINACA) were purchased from Cayman Chemicals (Ann Arbor, Michigan, USA). 5F-MDMB-P7AICA [Methyl-[2-(1-(5-fluoropentyl)-1H-pyrrolo[3,2-B]pyridine-3-carboxamido)-3,3-dimethylbutanoate], Cumyl-4CN-BINACA and Cumyl-PEGACLONE were obtained as “research chemicals” over the Internet. The internal standards AB-PINACA-D9, AB-FUBINACA-D9, ADBICA-D9, AKB48-D9, JWH-007-D9, JWH-015-D7, JWH-081-D9, JWH-122-D9, JWH-200-D5, JWH-210-D9, JWH-250-D5, MAM-2201-D5, PB-22-D9, RCS-4-D9, UR-144-D5 and XLR-11-D5 were obtained from Cayman Chemicals (Ann Arbor, Michigan, USA). JWH-018-D9 was from LGC Standards (Wesel; Germany), JWH-073-D9 from Chiron AS (Trondheim, Norway). Identities and purities (> 98%) of all reference standards not obtained from professional suppliers were confirmed by ^1^H and ^13^C nuclear magnetic resonance spectroscopy and GC–MS.

The alkaline carbonate buffer (pH 10) was prepared by mixing 534 mL of a sodium carbonate solution (0.1 mol/L) and 466 mL of sodium hydrogen carbonate solution (0.1 mol/L). For preparation of the “extraction mixture 1”, 990 mL of *n*-hexane and 10 mL of ethyl acetate (99/1, v/v) were mixed. For preparation of the “extraction mixture 2”, 800 mL of *n*-hexane and 200 mL of ethyl acetate (80/20, v/v) were mixed.

Mobile phase A consisted of water with 1% ACN, 0.1% formic acid and 1% of ammonium formate (200 mmol/L). Mobile phase B was ACN with 0.1% formic acid and 1% of ammonium formate (200 mmol/L).

### Homogenization of organs

One half g of liver and kidney was weighed with two decimal places. After addition of 10 µL of internal standard solution (10 µg/mL), five ceramic beads and 1 mL of ACN, the samples were homogenized for 3 min using a BeadBug Mictrotube Homogenizer from Süd-Laborbedarf GmbH (Gauting, Germany). Then, each sample was centrifuged at 16,550 g for 5 min and afterward stored overnight at 4 °C. The ACN supernatant was transferred into a sample tube and evaporated to dryness under a gentle stream of nitrogen at 60 °C. The residue was reconstituted in 1 mL of carbonate buffer. Extraction was performed in the same tube to make fat-rich residues available for analysis.

### Sample preparation

For analysis of cannabinoids, peripheral (femoral) and central blood (cardiac) was spiked with 10 µL of internal standards (THC-D3, 11-hydroxy-THC-D3: 10 µg/mL and carboxy-THC-D3: 25 µg/mL) before liquid–liquid extraction was performed using acetic acid (0.25 M) and *n*-hexane/ethyl acetate mixture (9/1, v/v). After subsequent shaking, the organic supernatant was separated and evaporated to dryness under a gentle stream of nitrogen. The extracts were derivatized (silylated) before analysis. Fentanyl and norfentanyl were analyzed in peripheral and cardiac blood after liquid–liquid extraction of postmortem samples. The samples were each spiked with 25 µL of internal standards (Fentanyl-D5 and Norfentanyl-D5: 25 µg/mL) before alkaline extraction. Pregabalin was analyzed after deproteinization according to Sorensen et al. [17] using vigabatrin as internal standard. For analysis of synthetic cannabinoids in cardiac blood and gastric content, sample volumes of 1 mL and 0.5 mL were used, respectively. After the sample was spiked with 10 µL of internal standards (10 µg/mL), 0.5 mL of carbonate buffer and 1.5 mL of “extraction mixture 1” were added. After 5 min of gentle mixing with an overhead shaker, the sample was centrifuged at 1500 g for 20 min. Subsequently, 1 mL of the organic supernatant was transferred to an HPLC vial and evaporated to dryness under a gentle stream of nitrogen at 40 °C. After addition of 1.5 mL of “extraction mixture 2”, the sample was treated as after addition of “extraction mixture 1”. Finally, the sample was reconstituted in 100 mL of mobile phase A/B (80/20, v/v).

The urine sample was prepared according to the procedure described by Franz et al. [18]. In brief, urine was extracted with ammonium formate and ACN fortified with deuterated internal standards of 18 synthetic cannabinoids and metabolites (salting-out assisted liquid–liquid extraction) after the incubation with glucuronidase. The mixture was shaken, centrifuged and the organic layer was transferred to a separate vial and evaporated. A positive control (containing metabolites of 5F-AB-PINACA, 5F-ADB, 5F-MDMB-P7AICA, AB-PINACA, ADB-FUBINACA, AMB, Cumyl-4CN-BINACA, Cumyl-PEGACLONE, FUB-AMB, MDMB-PINACA and others) consisting of 10 μL reference standard solution (1 μg/mL) and 10 μL of an extract of pooled authentic positive urine samples was prepared accordingly. For LC–MS/MS analysis, the residue was reconstituted in 200 μL of mobile phase A/B (50/50, v/v).

The preparation of the hair sample and the calibration and control samples followed the procedure described by Franz et al. [19]. The first hair segment (from proximal to distal: 0–3 cm) was used for the analyses. The hair sample was washed in 4 mL each of deionized water, acetone, and petroleum ether. After air drying, 27 mg of the hair sample was added to 1.5 mL methanol. The sample was cut into 1–2 mm pieces with scissors and 25 µL of internal standard solution (10 µg/mL) were added. Extraction was conducted by ultrasonication for 3 h. The methanolic extract was then transferred to a glass vial, evaporated, and reconstituted in mobile phase A/B (80/20, v/v) prior to LC–MS/MS analysis. Calibration samples (1.0, 2.5, 10, 50, 150 pg/mg) and control samples (blank, 2.0, 60, 120 pg/mg) were prepared accordingly (spiked with reference standards directly before the ultrasonication step).

### Instrumentation and conditions

The blood volatiles screening and determination of ethanol was carried out according to the guidelines for the determination of blood alcohol concentration (BAC) for forensic purposes [13]. The deproteinization step was carried out with trichloroacetic acid. The immunoassay was performed on an Olympus AU 400^®^ analyzer (Beckman Coulter, Brea, California, USA). The immunoassays were applied for the following substance groups: amphetamine and derivatives, benzodiazepines, cannabinoids, cocaine, opiates and methadone or metabolites (Cedia DAU^®^, Thermo Scientific, Waltham, Massachusetts, USA). The analysis was performed according to Köhler et al. [14] using an Olympus AU 400^®^ instead of a Thermo Indiko Plus Analyzer. GC–MS screening analysis was performed using mass selective detection (MSD) 5975C and a gas chromatograph 7890A equipped with autosampler and injector 7693 (Agilent Technologies, Santa Clara, California, USA). The extracted postmortem samples were separated chromatographically on an Optima-5 (30 m × 0.25 mm, 0.25 μm) column (Macherey–Nagel GmbH & Co. KG, Düren, Germany). The initial oven temperature was 70 °C followed by a 20 °C ramp to 320 °C and a subsequent 6-min holding time. After analysis, the resulting data files were evaluated manually and automatically using AMDIS with deconvolution and identification settings previously optimized for this type of analysis using the target library version of Maurer/Pfleger/Weber to identify drugs, active substances or pharmaceutical agents [15, 16].

The quantitative analysis of THC and metabolites was performed with GC–MS (see initial toxicological screening). The quantitative analysis of fentanyl/norfentanyl by LC–MS/MS was performed on an API 4000 MS/MS (AB Sciex, Framingham, Massachusetts, USA) with a TurboIon™ ionization source operating in the positive ionization mode. It was interfaced to an HPLC pump equipped with an autosampler (1100 series, Agilent Technologies) and a Phenomenex Luna C18 column (150 mm × 2 mm, 5 µm, Torrance, California, USA), the injection volume was 10 µL. LC–MS/MS parameters were according to Coopman et al. [20]. Pregabalin was determined by HILIC LC–MS/MS according to Sorensen et al. [17].

For synthetic cannabinoids, the LC–ESI–MS/MS system consisted of a QTrap 4000 triple quadrupole linear ion trap mass spectrometer fitted with a TurboIonSpray interface from Sciex (Framingham, Massachusetts, USA) and a Prominence HPLC system consisting of three LC-20ADsp isocratic pumps, a CTO-20AC column oven, a SIL-20AC autosampler, a DGU-20A3 degasser and a CBM-20A controller from Shimadzu (Kyoto, Japan). The temperature of the autosampler tray was set to 10 °C. Chromatographic separation of all substances was achieved using a Kinetex C18 column (50 × 2 mm, 5 µm) with guard column of the same material (4 × 2 mm), both from Phenomenex. The gradient of mobile phase A and B was as follows: initially, 20% of mobile phase B at a flow rate of 0.5 mL/min; held for 1 min; ramped to 60% of B within 1.5 min; ramped again to 65% of B within 1.5 min which was held for 1.5 min; ramped to 90% of B within 2.5 min and held for 2 min. Initial conditions were restored within 0.1 min and held for 2 min to re-equilibrate the system. The injection volume was 20 µL. The scheduled multiple reaction monitoring (sMRM) method included two transitions for each analyte and one transition for each internal standard. MRM transitions were recorded in a time window of ± 25 s around the expected retention time. The total cycle time in sMRM mode was 1.0 s including a pause time between MRM transitions of 5 ms. Considering the cycle time, the pause time and the maximum amount of overlapping sMRM transitions (*n* = 47), the minimum dwell time per transition was 20 ms. For each compound, the declustering potential (DP), the entrance potential (EP), the collision energy (CE), as well as the cell exit potential (CXP) were optimized.

The LC–ESI–MS/MS method applied for qualitative screening of synthetic cannabinoid metabolites in urine was reported in a previous publication [18]. If a synthetic cannabinoid showed MRM transitions from two corresponding metabolites or from one metabolite at a higher intensity, that synthetic cannabinoid was specified as “positive”. If MRM transitions of only one metabolite were detected at lower intensities, this synthetic cannabinoid was reported as “tentative”. The LC–ESI–MS/MS method applied for semi-quantitative determination of synthetic cannabinoids in hair was reported previously as well [19].

### Data analysis/interpretation

For synthetic cannabinoids and the main other drugs detected, on the basis of all data, a Toxicological Significance Score (TSS) was assigned in accordance to the methodology proposed by Elliott et al. [21]. According to the authors, the TSS ranges from 1 or ‘low’, i.e. alternative cause of death, to 3 or ‘high’, i.e. the NPS is cited as the cause of death or is likely to have contributed to the death or toxicity, even in presence of other drugs.

## Results

### Autopsy findings

The man (height: 179 cm, weight: 67 kg) showed no signs of external trauma, injection marks or fentanyl patches. At the section of internal organs, the heart (weight 440 g), displayed left ventricular hypertrophy and moderate coronary artery disease in two vessels (left descending and circumflex), in the absence of hemodynamically significant stenosis. Signs of atherosclerosis were also noted at the carotid arteries. Severe cerebral and pulmonary edema were observed, together with a gas inflation of the lungs (brain: 1515 g; lungs together: 2300 g approximately) [22]. The liver was slightly enlarged (weight: 1745 g) and showed the so-called “nutmeg” appearance. No additional pathological findings, including those non-specific but typically associated to intoxications such as hemolysis, congestion or urinary retention, were observed during autopsy.

### Analytical results of the case samples

The detailed results of the toxicological analyses in the different matrices are shown in Table 1. Fentanyl was detected in femoral blood at a concentration of 14 ng/mL (norfentanyl approx. 1.0 ng/mL) and pregabalin at a concentration of 3,200 ng/mL. Blood alcohol was not detected in peripheral blood. The immunoassays were negative for all substance groups of common drugs of abuse. In addition, the quantitative analyses of cannabinoids (THC, 11-hydroxy-THC, carboxy-THC) in blood were also negative.

**Table 1:** Results of qualitative and quantitative toxicological analyses of blood, urine, organs, gastric content, hair and water of the bucket

Compound	Femoral vein blood [ng/mL]	Cardiac blood [ng/mL]	Urine	Kidney [ng/mL]	Liver [ng/mL]	Gastric content	Bucket water [mg/L]	Hair* [pg/mg]
Fentanyl	14	23	+	n.a	n.a	–	1.7	770
Norfentanyl	~ 1.0	~ 1.0		n.a	n.a	–	n.a	11
Pregabalin	3,200	n.a	+	n.a	n.a	–	n.a	n.a
delta 9-THC	n.a	–	n.a	n.a	n.a	+	n.a	n.a
Caffeine, Nicotine/Cotinine	n.a	n.a	+	n.a	n.a	+	n.a	n.a
5F-AB-PINACA	n.a	–	( +)	–	–	–	–	–
5F-ADB	n.a	2.7	+	0.98	< 0.1	+	+	1,100
5F-AMB	n.a	–	( +)	–	–	–	–	78
5F-Cumyl-PICA	n.a	< 0.1	–	–	–	–	–	+
5F-MDMB-P7AICA	n.a	13	+	0.21	0.7	+	–	300
AB-CHMINACA	n.a	–	–	–	–	–	–	190
AB-FUBINACA	n.a	< 0.1	–	–	< 0.1	–	–	–
AB-PINACA	n.a	–	( +)	–	–	–	–	4
ADB-FUBINACA	n.a	< 0.1	+	–	+	–	–	68
AMB	n.a	–	( +)	–	–	–	–	–
Cumyl-4CN-BINACA	n.a	–	( +)	–	–	–	–	110
Cumyl-PEGACLONE	n.a	–	( +)	–	–	–	–	45
EG-018	n.a	1.9	–	+	+	+	–	11
FUB-AMB	n.a	+	+	+	+	+	–	> 250
MDMB-PINACA	n.a	–	( +)	–	–	–	–	–

n.a. not analyzed, + detected, ( +) tentatively detected, ~ approximately, – not detected (< LOD)

*In hair, the following additional drugs were detected: Amphetamine: 240 pg/mg, MDMA: 250 pg/mg, cocaine: 14 pg/mg, benzoylecgonine: 4.1 pg/mg, 6-acetyl morphine: 5.8 pg/mg, methadone: 1.1 pg/mg, tilidine: 11 pg/mg, nortilidine: 70 pg/mg, tramadol: 170 pg/mg, nordiazepam: 22 pg/mg, diazepam: 36 pg/mg, 5F-AMB-PICA: 1.9 pg/mg, 5F-ABICA: 12 pg/mg, ADB-CHMINACA: 55 pg/mg, Cumyl-4CN-BINACA: 110 pg/mg, Cumyl-PEGACLONE: 45 pg/mg, and PX-2: 150 pg/mg

The synthetic cannabinoids 5F-ADB, 5F-Cumyl-PICA, 5F-MDMB-P7AICA, AB-FUBINACA, ADB-FUBINACA, EG-018 and FUB-AMB were detected in cardiac blood. Concentrations above the calibration curve were extrapolated. Instability of the methyl esters 5F-ADB and 5F-MDMB-P7AICA in collected cardiac blood can be considered negligible, as the sample was frozen immediately. Six of the seven synthetic cannabinoids found in cardiac blood were also detected in kidney or liver. Metabolites of thirteen synthetic cannabinoids were identified in urine. The highest synthetic cannabinoid concentration in cardiac blood of approx. 13 ng/mL was found for 5F-MDMB-P7AICA. This synthetic cannabinoid or its metabolites were detected in all matrices. 5F-ADB and fentanyl were detected in the bucket water, indicating consumption via the bucket bong.

By far, the most synthetic cannabinoids (*n* = 17) were found in the hair sample indicating a frequent consumption of such compounds in the past. Some substances were only found in this matrix. This could be due to consumption of the respective synthetic cannabinoids in the past. However, external contamination by handling herbal blends or research chemicals containing the corresponding cannabinoids may have contributed to the findings [23].

Due to remarkable ion suppression of the internal standard, the results of gastric contents are only given qualitatively. The results of the synthetic cannabinoid metabolites in urine are qualitative as well as reference standards were not available for most of these compounds.

## Discussion

Here, we present a challenging case of suspected intoxication due to inhalation of drugs through a bucket bong. The suspicion of intoxication arose from the paraphernalia found at the death scene and the cause of death could not be explained only by the postmortem examination, since typical signs of chronic cardiovascular disease were found, albeit in the absence of acute or lethal modifications. The major toxicological findings in the blood were fentanyl, pregabalin, 5F-ADB, and 5F-MDMB-P7AICA. Their respective contributions to death are discussed below.

Fentanyl is a high-potency synthetic opioid, and, as seen with other narcotics, its therapeutic, toxic and fatal concentrations tend to overlap, depending on multiple variables including the level of tolerance as well as the route of administration. Typical mean maximum therapeutic concentrations of fentanyl in serum are in the range of 0.3–3.3 ng/mL within 72 hours after application of a fentanyl patch (dose 12 µg/hours to 100 µg/hours) [24]. After oral fentanyl intake, seven presumably opioid-tolerant adults showed signs of opioid intoxication and had fentanyl concentrations of 1.6 to 10 ng/mL in serum at hospital admission [25]. Postmortem levels of fentanyl are highly variable (from 9 to 30 ng/mL) with higher risks arising from unusual administration routes, as in the presented cases [11]. In three lethal cases after inhalation of the drug-reservoir of fentanyl patches heated on an alumina foil, postmortem fentanyl concentrations were 2.6 ng/mL in femoral blood, 6.0 ng/mL in cardiac blood, 41 ng/mL in urine [10], 5.8 ng/mL in femoral blood (subject 103 [26]), and 44 ng/mL in peripheral blood (case 8, patch doses 75 and 100 µg/hours [27]), respectively. In another case with suspected fentanyl patch smoking, fentanyl and norfentanyl concentrations were found to be 11 ng/mL and 3.2 ng/mL in femoral blood, 19 ng/mL and 5.3 ng/mL in cardiac blood, and 61 ng/mL and 330 ng/mL in urine [25]. In a comparative study, postmortem fentanyl blood concentrations were on average up to nine times higher than in vivo serum concentrations at the same dose [28]. The present fentanyl concentration of 14 ng/mL in femoral vein blood is above the typical range after therapeutic use, but might not have been lethal per se, as opioid tolerance and a postmortem concentration increase by a factor of 2–8 must be considered. The factor of 1.6 between cardiac and femoral blood is in good agreement with the literature, where factors of 1.6 and 2.7 have been described [29, 30]. Concentrations of norfentanyl are typically much lower than those of fentanyl after a fatal overdose, with mean peripheral blood concentrations of 2.0 ng/mL and 18 ng/mL, respectively [30]. In contrast, the steady-state plasma fentanyl and norfentanyl concentrations during continuous fentanyl infusion were more or less in the same range (1.8–4.6 ng/mL and 0.7–3.1 ng/mL, respectively) [31]. In the present case, the low concentration of norfentanyl (1.0 ng/mL) compared to fentanyl (14 ng/mL) is consistent with a very recent fentanyl uptake shortly before death. The fentanyl concentration in hair seems to be relatively high (above the 75th percentile in 60 authentic hair samples [32] and suggests the development of a certain tolerance to the toxic effects of fentanyl. Nevertheless, it cannot be sufficiently interpreted in terms of an assumed fentanyl consumption pattern, since smoked drugs can also lead to high concentrations in hair only via external contamination by side stream smoke only [33, 34]. Moreover, it is not possible to exclude a drug-free period with reduced tolerance before the fatal episode with the bucket bong.

Pregabalin is a prescription anticonvulsant and anxiolytic medication, which is commonly misused and abused, especially by people with opioid use disorder [35, 36]. Although gabapentinoids augment the effects of opioids and are commonly found in cases of deaths related to non-heroin-opioids, their causative relation to death is often not easy to judge [37]. There is no relevant postmortem redistribution reported for pregabalin in the literature [38] and, in the case here-in reported, the concentration of pregabalin can be assumed to be in the usual therapeutic range [39]. The contribution of pregabalin to death is therefore likely to be rather negligible, although synergistic effects cannot completely be ruled out.

5F-ADB has been reported to be a highly potent agonist at CB1 and CB2 receptors [6, 40] showing more than 290 times greater potency than delta-9-THC in a fluorometric assay of membrane potential [41]. Typical postmortem blood concentrations of 5F-ADB range from 0.01 to 2.2 ng/mL (median 0.34 ng/mL) in central blood and 0.01–0.77 (median 0.15) in peripheral blood (43 cases [42]). Comatose-fatal levels reported by Schultz et al. range from 0.11 to 1.92 ng/mL [39]. The concentration of 5F-ADB in the case here presented seems to be relatively high compared to other cases described in the literature [2] or analyzed in the institute in Freiburg (66 other postmortem cases, approximate concentrations in femoral or cardiac blood ranged from the limit of detection (LOD) to 14 ng/mL, median 0.15 ng/mL; the concentration of 2.7 ng/mL is within the upper 6% of measured concentrations). Nevertheless, concentrations have to be considered with caution, given that, due to passive postmortem redistribution, cardiac blood might show higher levels than peripheral blood, especially when, as in this case, the drug is present in the gastric content (although not quantified). Moreover, given the multiple synthetic cannabinoids found in the hair and the circumstantial data of frequent “herbal mixtures” abuse, the tolerance of the subject appears likely, and this could lead to even high concentrations in blood/serum in the absence of adverse events [6].

Since 5F-MDMB-P7AICA was not detected in the bucket water, it can be assumed that the consumption of 5F-MDMB-P7AICA occurred before smoking the bong. Nevertheless, the concentration of 5F-MDMB-P7AICA seems to be relatively high. Compared to other cases in our institute (4 other postmortem cases, approximate concentrations in femoral or cardiac blood ranged from the LOD to 7.5 ng/mL), the concentration of 18 ng/mL is more than twice the second highest concentration. Regarding the activity, the comparison of azaindole derivatives to their corresponding indazole or indole derivatives showed that azaindoles generally have lower affinity for CB receptors [43]. The cannabimimetic activity of 5F-MDMB-P7AICA showed comparatively low potency in a β-arrestin 2 recruitment assay at CB1 [44]. User forums on the Internet also report that azaindole derivatives are rather less active and tend to induce tolerance quickly. However, given the high concentration and the setting of polydrug consumption, 5F-MDMB-P7AICA might have enhanced the effects of 5F-ADB.

The assessment of the toxicological significance of the drugs in the present case appears challenging due to the co-consumption of multiple potent drugs and to a pattern of pre-existing chronic cardiovascular pathology emerged from the postmortem examination, which is usually absent at the age of the deceased. Coronary stenosis chronically reduces blood circulation to the heart, leading to a high susceptibility of the heart muscle to minor additional stress factors (e.g. physical or emotional stress, arrhythmia, transient reduction in the blood flow or vasospasm induced by a drug), which can ultimately result, in cases of luminal stenosis > 75%, in a sudden cardiac death [45]. In the present case, no acute myocardial infarction or other signs of acute cardiac death were noted and the postmortem findings are consistent with an incidental finding. On the other hand, signs of hepatic venous congestion usually related to a chronic cardiac failure emerged from the “nutmeg” appearance of the liver and from the hepatomegaly itself.

Opioids typically lead to death by ventilatory depressant effect and central nervous system hypoxia, which might occur particularly rapid and pronounced with fentanyl [46]. In the case presented here, the postmortem findings, particularly weight of the lungs and severe pulmonary edema, are consistent with the classic opioid-induced respiratory depression pattern or “heroin-lung” that has also been observed in fatal fentanyl intoxications. Opioids also bear a risk of cardiovascular toxicity and cardiac arrest, so that the use of some synthetic opioids is questioned in a setting of chronic coronary artery disease [47]. Fentanyl particularly has a dose-dependent effect on QT interval prolongation, causes bradyarrhythmia and asystole [48, 49] and, particularly in an illicit use and in combination with synergistic cardiotoxic drugs, could be related to sudden death [50].

Synthetic cannabinoids seem to have strong effects on the cardiovascular system, leading to tachycardia and hypertension more frequently then bradycardia and hypotension, as well as to several electrocardiographic changes [7, 51–53]. Synthetic cannabinoids have been linked also to myocardial infarction as well as to arrhythmia-related sudden cardiac deaths [2, 54], although a causative role has not been clearly established. On the other hand, synthetic cannabinoids can frequently induce respiratory depression and non-cardiogenic pulmonary edema, as shown in several intoxications and death cases [55–57]. To further complicate the interpretation, it has to be considered that the unusual administration route, through a bucket bong, could explain an increased and potentially unexpected toxicity and that the lung edema produced by drug-induced respiratory depression is virtually indistinguishable from that reported in patients who die of cardiogenic shock.

The pathological finding of severe brain edema, however, suggest a rather progressive hypoxia of the brain, preceding the cessation of cardiac output [49], as typically occurs in respiratory depression, while a sudden cardiac death might provide too little time for hypoxia to occur and to a severe edema to be formed. Moreover, repeated episodes of blood stasis in the lungs, as in cases of respiratory depressions, might lead to congestion in the right ventricle of the heart and, backwards, to the venous system of the liver, explaining its appearance.

Based on the comprehensive analysis of all postmortem (from the death scene investigation to the autopsy) and toxicological data, as well as on the primarily CNS depressant effects of the involved substances (particularly of fentanyl and 5F-ADB), the death appears to be caused by multiple drug toxicity superimposed on a pre-existing cardiac disease, with a rather rapid but progressive respiratory depression as the plausible mechanism of death. However, other hypotheses, e.g. a direct effect on pulmonary capillaries as suggested for heroin, or a cardiogenic shock could be formulated. Following the above, assessment of the toxicological significance of the fentanyl and 5F-ADB findings according to Elliott et al. resulted in a Toxicological Significance Score (TSS) of 3 (high, i.e. new psychoactive substance cited as cause of death or likely to have contributed to toxicity/death, even in presence of other drugs) [2, 21]. A TSS of 2 (medium, i.e., may have contributed to death, but other findings may be more important) could be assigned to the other synthetic cannabinoids including 5F-MDMB-P7AICA as well as pregabalin, given the fact that these substances are also associated with a psychoactive depressant action at the central nervous system.

The chronic changes of the cardiovascular system could not be considered as an alternative cause of death, but might have had a role, by decreasing the possibility of the subject to increase the cardiac output in response to the severe CNS depression and to ultimately tolerate the toxic effects of the drugs.

## Conclusion

In the present case, a bucket bong, retrieved on the crime scene, was likely used to consume fentanyl, obtained by a patch, and the synthetic cannabinoid 5F-ADB. 5F-MDMB-P7AICA was consumed previously. The assumingly weak cannabimimetic effect of 5F-MDMB-P7AICA might have prompted the man to subsequently change the mode of administration, switching to bucket smoking, which facilitates the quick uptake of high amounts of drugs and usually results in a very rapid onset of drug effects. On the basis of a comprehensive analysis of data, the cause of death could be primarily attributed to a respiratory depression due to the effects of fentanyl and synthetic cannabinoids (in particular 5F-ADB) in a subject suffering from chronic heart disease. This case of an acute intoxication and fatality demonstrates that use of opioids in combination with synthetic cannabinoids might be particularly dangerous. The rather unusual mode of administration as well as the multi-drug consumption and the pre-existing conditions might have played a role in the death.
